# Sources of Dietary Fiber Affect the SCFA Production and Absorption in the Hindgut of Growing Pigs

**DOI:** 10.3389/fnut.2021.719935

**Published:** 2022-01-10

**Authors:** Yu Bai, Xingjian Zhou, Jinbiao Zhao, Zhenyu Wang, Hao Ye, Yu Pi, Dongsheng Che, Dandan Han, Shuai Zhang, Junjun Wang

**Affiliations:** ^1^State Key Laboratory of Animal Nutrition, College of Animal Science and Technology, China Agricultural University, Beijing, China; ^2^State Key Laboratory of Biological Feed, Ministry of Agriculture and Rural Affairs, Boen Biotechnology Co. Ltd., Ganzhou, China; ^3^College of Animal Science and Technology, Jilin Agricultural University, Changchun, China

**Keywords:** fiber sources, *in vivo-vitro* method, SCFA production and absorption, hindgut fermentation, growing pigs

## Abstract

Effects of different dietary fiber (DF) sources on short-chain fatty acids (SCFA) production and absorption in the hindgut of growing pigs were studied by an *in vivo–vitro* (ileal cannulated pigs and fecal inoculum-based fermentation) method. Thirty-six cannulated pigs (body weight: 48.5 ± 2.1 kg) were randomly allocated to 6 treatments containing the same DF content (16.5%), with either wheat bran (WB), corn bran (CB), sugar beet pulp (SBP), oat bran (OB), soybean hulls (SH), or rice bran (RB) as DF sources. Pigs were allowed 15 days for diet adaptation, and then, fresh ileal digesta and feces were collected to determine SCFA concentration which was normalized for food dry matter intake (DMI) and the hindgut DF fermentability. Fecal microbiota was inoculated into the freeze-dried ileal digesta samples to predict the ability of SCFA production and absorption in the hindgut by *in vitro* fermentation. The SH group had the largest concentration of total SCFA and propionate in ileal digesta and fecal samples of growing pigs (*p* < 0.05). Nonetheless, the predicted acetate, total SCFA production, absorption in the SBP group were the highest (*p* < 0.01), but the lowest in the OB group (*p* < 0.01) among all groups. Even SBP and OB group had a similar ratio of soluble DF (SDF) to insoluble DF (IDF). The CB group had high determined ileal and fecal butyrate concentration but the lowest butyrate production and absorption in the hindgut (*p* < 0.01). Overall, the source of DF had a great impact on the hindgut SCFA production and absorption, and SBP fiber had a great potential to increase hindgut SCFA production and absorption.

## Introduction

As one of the major dietary components, dietary fiber (DF) could hardly be digested in the foregut due to the lack of corresponding enzymes but could be fermented by microbiota in the gastrointestinal tract (GIT) (1). Short-chain fatty acids are the main metabolites of DF fermentation, including acetate, propionate, and butyrate. Many reports indicated that SCFA was helpful to prevent intestinal diseases, promote proliferation of GIT tissue and the absorption of minerals, and reduce the level of cholesterol in the blood (2, 3). Besides, SCFA produced in the hindgut was an important energy source for humans and pigs. It had been reported that 10–13% of the energy requirement in humans and pigs were supplied by SCFA (4, 5).

Wheat bran (WB), corn bran (CB), sugar beet pulp (SBP), oat bran (OB), soybean hulls (SH), and rice bran (RB) have been widely used in foods and feeds as ubiquitous fibrous ingredients. DF is the core component of these fibrous ingredients, and the ability of DF to produce SCFA in the GIT is the essential fermentation characteristic of these fibrous ingredients. Fermentation characteristic of DF in fibrous ingredients has been explored in many trials. But these researches had just concluded that the fermentation of CB, SB, and WB had a preference for butyrate production in weaned pig's hindgut, or WB was easier to be fermented into SCFA than OB (6, 7). However, the SCFA concentration in these researches could not reveal the true SCFA production ability of DF, because DF in commercial diets was not only origin from fibrous ingredients but also the other feedstuffs, such as corn, soybean meal, and so on.

Furthermore, the SCFA in feces was assumed as the ability of SCFA production in the hindgut in previous human and animal research (8, 9). Short-chain fatty acids existed in digesta and feces could not represent the true production ability of SCFA, but the remnant of SCFA absorbed in the hindgut. Short-chain fatty acids in the hindgut were absorbed by epithelium and diffused into the portal vein, and the SCFA excreted in the feces only accounted for 5% of SCFA production in the hindgut (10, 11). Besides, short-chain fatty acids consumed by bacteria cannot be neglected, because bacteria were known to cross-feeding on SCFA (12). An *in vivo–vitro* method was used to estimate the amount of SCFA production and absorption in the hindgut (13). Ileal digesta was served as a substrate and inoculated with fecal microbiota to predict the SCFA production in the hindgut (simulate the fermentation activity in the hindgut). Finally, short-chain fatty acids in fresh ileal digesta, fecal sample and production *in vitro* were applied to calculate the amount and proportion of SCFA absorbed in the hindgut (14). Notwithstanding, the effect of DF solubility on the SCFA production and absorption in the hindgut had been investigated in the previous research (15), but the interference of other sources of DF and the interactions between the different sources of DF were neglected.

This study used the growing pig model to predict the impact of different sources of DF on SCFA production and absorption in the hindgut by an *in vivo–vitro* method.

## Materials and Methods

This study followed the Laboratory Animal Welfare and Animal Experimental Ethical Inspection Committee in China Agricultural University (AW32110202-2). The *in vivo* trial was conducted at the Swine Research Unit of China Agricultural University (Beijing, China).

### Experimental Diets and Pigs

WB, CB, SBP, OB, SH, and RB were selected as the only DF source for each treatment. The total dietary fiber (TDF) in the diets was contained the same level (16.5%) through modulating the inclusion level of fibrous ingredients. The result of diet chemical compositions and the feed ingredients were shown in Table 1. All diets were formulated to provide enough or exceed vitamins and minerals for experimental animals based on nutrient requirements of swine (16). 0.3% chromic oxide was added as indigestible marker for the determination of digestibility. Thirty-six (Duroc × Landrace × Large White) crossbred barrows (initial body weight of 48.5 ± 2.1 kg) were operated with a T-cannula in the distal ileum, ~5 cm cranial to the ileocecal sphincter. All pigs were housed in individual stainless-steel metabolism crates (1.3 × 0.8 × 0.7 m) and allowed 14 days for recovery after surgery. Surgical and nursing procedures were following the Stein's research (17).

**Table 1:** Ingredients and nutrient compositions of the diets.

	**Diets^a^**					
	**WB**	**CB**	**SBP**	**OB**	**SH**	**RB**
**Ingredient (%)**						
Corn starch	35.08	46.36	45.95	34.63	45.13	21.43
Soy protein isolate	12.00	12.00	14.00	12.00	14.00	11.00
WB	35.70	–	–	–	–	–
CB	–	25.00	–	–	–	–
SBP	–	–	22.50	–	–	–
OB	–	–	–	35.80	–	–
SH	–	–	–	–	23.50	–
RB	–	–	–	–	–	50.00
Soy oil	30.00	30.00	30.00	30.00	30.00	30.00
Sucrose	10.00	10.00	10.00	10.00	10.00	10.00
Limestone	1.10	0.55	–	0.50	–	0.50
Dicalcium-phosphate	0.80	0.60	2.20	1.80	2.10	1.80
Cr_2_O_3_	0.30	0.30	0.30	0.30	0.30	0.30
NaCl	0.45	0.45	0.45	0.45	0.45	0.45
K_2_CO_3_	0.30	0.30	0.30	0.30	0.30	0.30
MgO	0.10	0.10	0.10	0.10	0.10	0.10
*L*-lysine-HCl	0.40	0.48	0.35	0.42	0.30	0.32
DL-Methionine	0.12	0.16	0.15	–	0.12	0.12
L-Threonine	0.15	0.20	0.20	0.20	0.20	0.18
Premix^b^	0.50	0.50	0.50	0.50	0.50	0.50
**Nutrient (%, DM)**						
GE (MJ/kg DM)	1.82	1.85	1.82	1.80	1.83	1.81
CP	12.80	13.32	13.76	14.28	13.33	15.22
EE	2.24	1.83	0.73	5.80	1.59	1.57
TDF	16.41	16.53	16.58	16.54	16.42	16.32
SDF	1.44	0.74	6.54	7.50	3.10	1.45
IDF	14.97	15.79	10.04	9.04	13.32	14.87
SDF/IDF (%)	9.62	4.69	62.05	82.96	23.27	9.75
NDF	14.07	17.93	13.61	17.96	18.11	15.06
ADF	3.87	4.64	5.97	3.15	10.80	5.96
Cellulose	3.06	3.92	4.32	2.00	9.29	1.03
Hemicellulose	10.20	13.29	7.64	14.81	7.31	9.09

a *WB, wheat bran; CB, corn bran; SBP, sugar beet pulp; OB, oat bran; SH, soybean hulls; RB, rice bran*.

b *Vitamin A, 5512 IU/kg; vitamin D3, 2200 IU/kg; vitamin E, 64 IU/kg; vitamin K3, 2.2 mg/kg; vitamin B12/kg, 27.6 ug/kg; riboflavin, 5.5 mg/kg; pantothenic acid, 13.8 mg/kg; niacin, 30.3 mg/kg; choline chloride, 551 mg/kg; Mn (MnO), 40 mg/kg; Fe (FeSO_4_·H_2_O), 100 mg/kg; Zn (ZnO), 100 mg/kg; Cu (CuSO_4_·5H_2_O), 100 mg/kg; I (KI), 0.3 mg/kg; Se (Na_2_SeO_3_), 0.3 mg/kg. (The concentration of vitamins and trace minerals was showed in diet content style) GE, gross energy; CP, crude protein; EE, ether extract; TDF, total dietary fiber; SDF, soluble dietary fiber; IDF, insoluble dietary fiber; NDF, neutral detergent fiber; ADF, acid detergent fiber*.

### Experimental Design and Sample Collection *in vivo* Trial

A total of 36 pigs were randomly allocated to the six dietary treatments (six pigs/diet) and provided water *ad libitum*. The estimated requirement for maintenance energy (i.e., 197 Kcal ME/ kg body weight^0.6^) was provided with pigs for 3 times (16). Two equivalent daily meals were provided at 08:30 and 16:30. The animal experiment lasted for 21 days, including the first 15 days for diet adaptation, and all feces were collected on the 16th, 17th, and 18th day. Ileal digesta was collected on the last 3 days (19th, 20th, and 21th day) and collected for 16 hours per day until no digesta outflows. A total of 36 samples of ileal digesta were collected for *in vitro* fermentation assay. Fresh feces were collected by the rectal palpation technique and stored at −80°C for fecal SCFA determination. Feces naturally excreted by pigs were collected in sterile plastic bags immediately and saved in −20°C condition. For ileal digesta collection, sterile polyethylene bags were used to collect ileal digesta by attaching barrel of the cannula. Bags were replaced every 25 min unless filled with ileal digesta and stored at −20°C. The first bag of fresh digesta was sampled and stored at −80°C immediately for ileal SCFA determination. At the end of the animal trail, all samples were pooled based on pig and collection date. Ileal digesta and feces were freeze-dried, mixed sufficiently, and stored at −20°C.

### *In vitro* Fermentation Assay

The procedure of ileal digesta fermentation and fecal inoculum preparation was based on the previous studies (14, 18). In brief, fresh feces were collected from six growing pigs [50 kg bodyweight approximately and fed with commercial grower diet (Supplementary Table 1)] without the use of antibiotics in the last 3 months before feces collection. Then, the feces were homogenized with sterile 0.1 M PBS at pH 7 (1:5, w/v) and filtered with four layers of sterile gauzes to serve as the inoculum. Then, 500 mg of freeze-dried ileal digesta (DMI) samples collected *in vivo* trail was accurately weighed into the serum bottle and added with 25 mL inoculum. The empty bottles were incubated with 25 mL of phosphate buffer and served as blank control. The serum bottles were flushed with CO_2_ and capped immediately after inoculation and then transported to a prewarmed (37°C) incubator for 38 h. All the procedures were conducted in a condition filled with CO_2_ at 37°C. Four replicate bottles were prepared for substrate or blank, one for SCFA determination and the other for the determination of dry matter (DM) fermentability. After incubation, the fermentation broth was centrifuged at 15,000 g for 12 min at 4°C. The fermentation broth supernatant was collected after centrifugation and stored at −80°C until analyzed for SCFA concentration. The DM of the unfermented residue was determined by drying them at 65°C until constant weight reached after sterilization.

### Chemical Analyses

The diets, ileal digesta, and feces were analyzed in duplicate for DM, chromium, SDF, IDF, neutral detergent fiber (NDF), acid detergent fiber (ADF), acid detergent lignin (ADL), cellulose, and hemicellulose. The diets were also determined for gross energy (GE), ether extract (EE), and cross-protein (CP). The DM (Method 934.01), EE (Method 920.39), crude protein (CP) (Method 990.03), chromium (Method 990.08), soluble DF (SDF), and insoluble DF (IDF) (Method 991.43) in the diets were also analyzed (19). NDF and ADF were determined by the method of Van Soest et al. (20). Hemicellulose was seen as the difference between NDF and ADF, and cellulose was the difference between ADF and ADL.

The concentration of SCFA in ileal digesta, fecal samples, and fermentation supernatants was determined by previous method (21). The sum of the acetate, propionate, and butyrate was seen as total SCFA, and the branch-chain fatty acids were not considered in this research.

### Calculation and Statistical Analysis

#### *In vivo* Assay

The determined hindgut fermentability was calculated as follows:


$$\begin{array}{l} {\text{Determined hindgut fermentability}}_{in\;vivo}\;\left(\%\right)\\ \;=\left(1-\left(\left({\text{DM}}_{\text{F}}÷{\text{DM}}_{\text{I}}\right)\times \left({\text{T}}_{\text{I}}÷{\text{T}}_{\text{F}}\right)\right)\right)\times 100 \end{array}$$

where T_I_ and T_F_ are the chromic oxide contents (g/kg DM) in ileal digesta and fecal samples; DM_F_ and DM_I_ are the contents of DM (g/kg) in fecal samples or ileal digesta, respectively.

The concentration of SCFA in ileal digesta and fecal samples (normalized for DMI) was calculated according to the research of Montoya et al. (14).


$$\begin{array}{l} \text{Normalized SCFA concentration }\left(\text{mmol}/\text{kg DMI}\right)\\ \;=\text{SCFA concentration }\left(\text{mmol}/\text{kg DM}\right)\times \left({\text{T}}_{\text{D}}÷{\text{T}}_{\text{F}/\text{I}}\right) \end{array}$$

where T_D_, T_F_, and T_I_ are the chromic oxide content (g/kg DM) in the diet, fecal samples, and ileal digesta, respectively.

#### *In vitro* Fermentation Assay

The fermentability of DM and the production of SCFA were seen as the predicted hindgut fermentability of DM, and the production of SCFA in the hindgut was calculated as follows:


$$\begin{array}{l} {\text{Predicted hindgut fermentability}}_{in\;vitro}\;\left(\%\right)\\ \;=\left(\text{D}{\text{M}}_{\text{b}}-\left(\text{D}{\text{M}}_{\text{a}}-\left(\text{D}{\text{M}}_{\text{blank initial}}+\text{D}{\text{M}}_{\text{blank final}}\right)/2\right)\right)/\\ \text{ D}{\text{M}}_{\text{b}}\times 100\\ \text{Production of SCFA during fermentation}\\ \;\left(\text{mmol}/\text{kg DM incubated}\right)\\ =\left(\text{SCF}{\text{A}}_{\text{sample}}-\left(\frac{\left(\text{SCF}{\text{A}}_{\text{blank initial}}+\text{SCF}{\text{A}}_{\text{blank final}}\right)}{2}\right)\right)/\\ \text{ sample weight }\left(\text{g DM}\right)\;\times 1,000 \end{array}$$

where DM_b_ and DM_a_ are the DM (mg) of the ileal digesta either before or after fermentation. DM_blankinitial_, DM_blankfinal_, SCFA_blankinitial_, and SCFA_blankfinal_ are the DM (mg) and the SCFA (mmol) in the blank bottle before (initial) and after (final) fermentation, respectively (22).

The predicted SCFA production was calculated based on the *in vivo–vitro* combining result (ileal cannulated pig and mimic hindgut fermentation). The predicted hindgut SCFA production was calculated as following equations (14):


$$\begin{array}{l} \text{Predicted hindgut SCFA production }\left(\text{mmol}/\text{kg DMI}\right)\\ =\text{SCFA produced by fermentation}\\ \;\left(\text{mmol}/\text{kg ileal digesta DM incubated}\right)\\ \;\times \text{Ileal DM flow }\left(\text{kg DM}/\text{kg DMI}\right) \end{array}$$

where ileal DM flow (g/kg DMI) is the ileal flow of DM.

Short-chain fatty acids absorption were predicted by the combined results of SCFA entering and producing in the hindgut (14):


$$\begin{array}{l} \text{Amounts of SCFA absorbed in the hindgut }\left(\text{mmol/kg DMI}\right)\\ =in\;vitro\text{ predicted hindgut SCFA production }(\text{mmol/}\\ \text{ kg DMI})\text{+ileal SCFA concentration }\left(\text{mmol/kg DMI}\right)\\ \text{ - fecal SCFA concentration }\left(\text{mmol/kg DMI}\right)\\ \text{The extent of SCFA absorbed in the hindgut }(\%)\;\\ \text{= amount of SCFA absorbed in the hindgut }(\text{mmol/}\\ \text{ kg DM intake})\text{/}(in\;vitro\text{ predicted hindgut SCFA production}\\ \;(\text{mmol/kg DMI})\text{+ ileal SCFA concentration}\\ \;(\text{mmol/kg DMI})) \end{array}$$

#### Statistical Analysis

Statistical analysis was conducted by SPSS software package (SPSS v. 20.0, SPSS Inc, Chicago, IL, USA). Kruskal–Wallis test was performed to test the impact of different fiber sources on the data of *in vivo* trail and *in vitro* fermentation trail. The ileal and fecal data were compared by Mann–Whitney test. *p* < 0.05 was considered significant.

## Results

### SCFA Concentration in Ileal Digesta and Feces

Pigs fed diets containing CB and SH had the highest (*p* < 0.01) normalized ileal acetate among all groups (Table 2), and the SH group also had the highest (*p* < 0.01) normalized ileal total SCFA, propionate, and butyrate. In contrast to the SH group, the normalized ileal total SCFA, acetate, and propionate in the OB group were the lowest (*p* < 0.01) among all groups. The WB group had the lowest (*p* < 0.01) normalized ileal butyrate as well. The normalized total SCFA and propionate in the fecal samples of SH group were higher (*p* < 0.05) than that of SBP, WB, OB, and RB. The normalized acetate concentration in fecal samples was higher (*p* < 0.05) than that in ileal digesta for all groups. Furthermore, the determined ileal and fecal SCFA in the SH treatment were the highest (*p* < 0.05) among all groups except ileal acetate (Supplementary Table 2). However, the RB group showed the lowest (*p* < 0.05) determined fecal SCFA concentration.

**Table 2:** Normalized ileal and fecal SCFA concentration for ileal cannulated pigs fed diets containing different sources of fiber (*n* = 6 per group).

	**Diets^1^**							
**Items**	**WB**	**CB**	**SBP**	**OB**	**SH**	**RB**	**SEM**	***P*** **-value**
**Normalized ileal SCFA (mmol/kg DMI) (** ***in vivo*** **)**								
Acetate	150.70^ab^	263.62^a^	256.71^ab^	125.74^b^	275.68^a^	247.88^ab^	23.02	<0.01
Propionate	41.51^b^	44.83^b^	15.62^b^	3.84^b^	129.66^a^	27.63^b^	8.58	<0.01
Butyrate	2.27^c^	8.15^abc^	7.09^bc^	15.26^ab^	17.60^a^	7.01^bc^	1.74	<0.01
Total SCFA^2^	194.48^bc^	316.59^ab^	279.42^abc^	144.85^c^	422.94^a^	282.52^abc^	25.33	<0.01
**Normalized fecal SCFA (mmol/kg DMI) (** ***in vivo*** **)**								
Acetate	94.16	119.05	86.67	74.62	114.23	97.12	10.08	> 0.05
Propionate	38.16^b^	59.90^ab^	31.20^b^	29.02^b^	78.82^a^	35.53^b^	6.97	<0.01
Butyrate	21.93	26.77	21.76	23.87	42.20	11.39	6.18	> 0.05
Total SCFA	154.25^b^	205.72^ab^	139.63^b^	127.52^b^	235.25^a^	144.04^b^	22.20	<0.05
**Statistical analysis for ileal vs. fecal SCFA concentration^3^**								
*P*_Acetate_	<0.05	<0.05	<0.05	<0.05	<0.01	<0.01		
SEM	18.89	24.78	26.44	11.33	10.41	7.43		
*P*_Propionate_	> 0.05	> 0.05	<0.05	<0.01	<0.05	> 0.05		
SEM	15.06	15.01	2.14	3.72	6.49	4.21		
*P*_Butyrate_	<0.05	<0.01	<0.05	<0.05	<0.05	<0.01		
SEM	3.94	2.72	4.21	4.25	6.29	2.35		
*P*_Total_ _SCFA_	> 0.05	<0.05	<0.05	> 0.05	<0.05	<0.01		
SEM	29.95	35.11	31.99	17.34	21.86	6.33		

1 *WB, wheat bran; CB, corn bran; SBP, sugar beet pulp; OB, oat bran; SH, soybean hulls; RB, rice bran. Normalized SCFA (mmol/kg DMI) = SCFA (mmol/kg DM) × (chromic oxide in the diets ÷ chromic oxide content in feces or ileal digesta)*.

2 *Total SCFA = acetate + propionate + butyrate*.

3 *P_Acetate_, P_Propionate_ P_Butyrate_, and P_Total_ _SCFA_ are the p-values for comparing ileal and fecal acetate, propionate, butyrate, and total SCFA, respectively*.

a,b,c *Values in the same row with different letter superscripts means significant difference (p < 0.05)*.

### Short-Chain Fatty Acids Production During *in vitro* Fermentation and Predicted SCFA Production in the Hindgut

The production of total SCFA, acetate, and propionate *in vitro* was the highest (*p* < 0.01) in SBP group but the lowest (*p* < 0.01) in OB group, and also predicted total SCFA, acetate, and propionate production (*p* < 0.01) in the hindgut (Table 3). Besides, the propionate and butyrate production *in vitro* and predicted production in the hindgut of the CB group were the lowest (*p* < 0.01) among all groups. Butyrate production in WB group was the highest (*p* < 0.01), but there was no difference in predicted production of butyrate in hindgut among the WB, SBP, OB, SH, and RB groups.

**Table 3:** Predicted SCFA production and absorption in hindgut (*in vivo–vitro* assay) of pigs fed the diets with different sources of fiber (*n* = 6 per group).

**Items**	**Diets^1^**							
	**WB**	**CB**	**SBP**	**OB**	**SH**	**RB**	**SEM**	***P*** **-value**
**SCFA production by** ***in vitro*** **fermentation method (mmol/kg DM incubated) (*****in vitro*** **method)**								
Acetate	4047.85^ab^	3520.92^bc^	5035.66^a^	2409.29^cd^	1996.28^d^	3476.45^bc^	232.34	<0.01
Propionate	562.13^b^	226.33^c^	1196.25^a^	598.56^bc^	231.55^c^	640.17^b^	74.60	<0.01
Butyrate	1160.61^a^	161.65^c^	699.00^b^	735.99^b^	697.51^b^	599.07^b^	72.06	<0.01
Total SCFA^2^	5770.59^ab^	3908.90^cd^	6930.91^a^	3743.84^cd^	2925.34^d^	4715.70^bc^	255.60	<0.01
**Predicted SCFA production in hindgut (mmol/kg DM intake) (*****in vivo–vitro*** **method)**								
Acetate	909.43^bcd^	1008.98^ab^	1404.80^a^	539.71^d^	598.39^cd^	999.82^abc^	67.96	<0.01
Propionate	125.97^bc^	64.65^c^	333.16^a^	134.30b^c^	69.82^c^	185.24^b^	18.36	<0.01
Butyrate	261.34^a^	46.32^b^	198.42^a^	164.34^a^	209.13^a^	170.87^a^	20.77	<0.01
Total SCFA	1296.74^bc^	1119.96^bcd^	1936.38^a^	838.36^d^	877.34^cd^	1355.93^b^	75.65	<0.01
**Predicted SCFA absorption in the hindgut (mmol/kg DM intake) (*****in vivo–vitro*** **method)**								
Acetate	965.96^bc^	1153.55^b^	1574.84^a^	590.83^c^	759.85^c^	1150.58^b^	66.27	<0.01
Propionate	129.32^bc^	49.58^c^	317.58^a^	109.12^bc^	120.66^bc^	177.34^b^	21.39	<0.01
Butyrate	241.68^a^	27.70^b^	183.75^a^	155.74^a^	184.53^a^	166.49^a^	20.02	<0.01
Total SCFA	1336.96^bc^	1230.83^bcd^	2076.17^a^	855.69^d^	1065.04^cd^	1494.41^b^	70.28	<0.01
**Predicted apparent SCFA absorption in hindgut (%) (*****in vivo–vitro*** **method)**								
Acetate	91.08^ab^	90.55^ab^	94.79^a^	88.85^b^	86.95^b^	92.11^ab^	0.95	<0.01
Propionate	78.01^ab^	43.98^c^	91.06^a^	77.30^ab^	60.11^bc^	82.60^ab^	4.57	<0.01
Butyrate	91.76^a^	50.18^b^	89.60^a^	86.46^a^	81.60^a^	92.75^a^	3.07	<0.01
Total SCFA	89.75^ab^	85.64^bc^	93.75^a^	86.96^abc^	82.08^c^	91.12^ab^	1.36	<0.01

1 *WB, wheat bran; CB, corn bran; SBP, sugar beet pulp; OB, oat bran; SH, soybean hulls; RB, rice bran*.

2 *Total SCFA = acetate + propionate + butyrate*.

a−d *Values in the same row with different letter superscripts means significant difference (p < 0.05)*.

### Predicted SCFA Absorption in the Hindgut

The predicted total SCFA, acetate, propionate, and butyrate absorption in the hindgut were the highest (*p* < 0.01) in SBP group (Table 3). The lowest (*p* < 0.01) amount of acetate but the highest (*p* < 0.01) amount of butyrate absorption was found in the hindgut of pigs fed OB-containing diet. The lowest (*p* < 0.01) absorption of butyrate and propionate also occurred in CB group.

Pigs in the SBP group had the higher (*p* < 0.01) predicted apparent total SCFA, acetate, and propionate absorption in the hindgut than SH group (Table 3). The predicted apparent absorption of total SCFA, propionate, and butyrate in SBP group was also higher (*p* < 0.01) than that of CB group. In addition, the predicted apparent absorption of propionate and butyrate in CB group was the lowest (*p* < 0.01).

### Hindgut Fermentability of Fiber for Growing Pigs Fed Diets Containing the Different Sources of Fiber

The SBP, OB, and SH group had the higher (*p* < 0.01) hindgut NDF fermentability than WB, CB, and RB group, and there was no difference in the hindgut fermentability of NDF among the SBP, OB, and SH groups (Table 4). Besides, the hindgut fermentability of SDF and IDF had the same trend as that of NDF among all groups. The hindgut fermentability of ADF in WB and RB group was the lowest (*p* < 0.01). The CB and RB group had the lower (*p* < 0.01) hindgut fermentability of cellulose and hemicellulose than SBP and OB group.

**Table 4:** Determined apparent hindgut fiber fermentability for ileal cannulated pigs fed the diets with different sources of fiber (*n* = 6 per group).

**Items^2^ (%)**	**Diets^1^**							
	**WB**	**CB**	**SBP**	**OB**	**SH**	**RB**	**SEM^2^**	***P*** **-value**
NDF	33.72^b^	27.93^b^	66.13^a^	72.99^a^	64.85^a^	24.58^b^	3.01	<0.01
ADF	8.70^d^	28.72^c^	68.41^a^	52.04^b^	62.29^ab^	7.00^d^	2.21	<0.01
TDF	38.69^bc^	29.83^c^	78.10^a^	74.12^a^	72.63^a^	45.43^b^	3.14	<0.01
SDF	63.02^b^	54.67^b^	94.75^a^	95.07^a^	84.21^a^	60.45^b^	3.25	<0.01
IDF	36.44^b^	28.70^b^	69.15^a^	63.00^a^	70.29^a^	44.09^b^	3.46	<0.01
Cellulose	9.41^d^	31.59^c^	86.26^a^	63.16^b^	64.08^b^	27.69^c^	3.01	<0.01
Hemicellulose	46.66^bc^	27.62^c^	63.17^ab^	52.46^a^	69.32^a^	37.12^c^	3.82	<0.01

1 *WB, wheat bran; CB, corn bran; SBP, sugar beet pulp; OB, oat bran; SH, soybean hulls; RB, rice bran*.

2 *ND, neutral detergent fiber; ADF, acid detergent fiber; TDF, total dietary fiber; SDF, soluble dietary fiber; IDF, insoluble dietary fiber*.

a−d *Values in the same row with different letter superscripts means significant difference (p < 0.05)*.

### Determined and Predicted Hindgut DM Fermentability for Growing Pigs Fed Diets Containing Different Sources of Fiber

The SBP, OB, and SH group were found to have the higher (*p* < 0.01) hindgut DM fermentability than WB, CB, and RB, and there was no difference in the hindgut fermentability of DM among the SBP, OB, and SH group (Table 5). Furthermore, the SBP group had the highest (*p* < 0.01) predicted hindgut DM fermentability followed by the OB group, and the predicted hindgut DM fermentability for WB and CB groups was the lowest (*p* < 0.01). The predicted hindgut DM fermentability was higher (*p* < 0.05) than the determined hindgut DM fermentability in OB group, whereas there were no differences in the other groups.

**Table 5:** Determined and predicted hindgut DM fermentability for ileal cannulated pigs fed the diets with different sources of fiber (*n* = 6 per group).

**Items**	**Diets^1^**							
	**WB**	**CB**	**SBP**	**OB**	**SH**	**RB**	**SEM^2^**	***P*** **-value**
**Determined and predicted hindgut DM^2^ fermentability**								
Determined fermentability, % (*in vivo*)	35.76^b^	36.15^b^	66.34^a^	56.58^a^	60.28^a^	33.60^b^	3.09	<0.01
Predicted fermentability, % (*in vivo–in vitro*)	35.66^e^	31.17^e^	73.21^a^	67.00^b^	54.76^c^	41.03^d^	0.86	<0.01
**Comparison of determined and predicted DM fermentability**								
$P_{\text{hindgutfermentability}}^{3}$	0.97	0.31	0.26	0.02	0.23	0.06		
SEM	1.47	2.18	3.22	1.77	1.82	1.39		

1 *WB, wheat bran; CB, corn bran; SBP, sugar beet pulp; OB, oat bran; SH, soybean hulls; RB, rice bran*.

2 *DM, Dry matter*.

3 *P_hindgutfermentability_ is the p-value for comparing the determined and predicted hindgut DM fermentability*.

a−d *Values in the same row with different letter superscripts means significant difference (p < 0.05)*.

## Discussion

**Figure 1:**
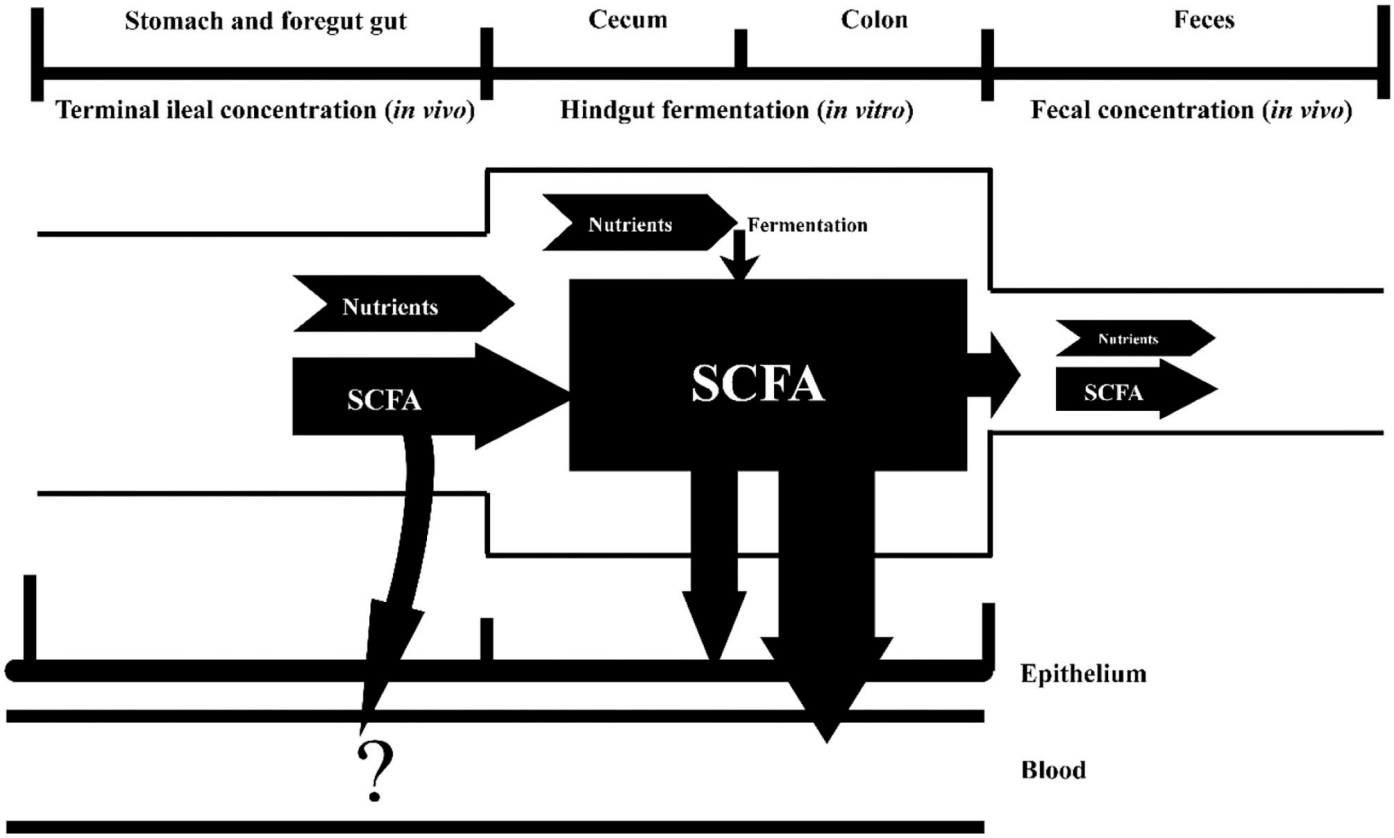
An *in vivo–vitro* method to determine SCFA production and absorption in the hindgut. The “?” represents the SCFA absorption in the foregut, which was not a measurement in this research.

This research investigated the impact of different DF sources on the predicted SCFA production and absorption in the hindgut using pig model. Recently, the probiotic characteristics of DF have attracted huge amounts of interest, and WB, CB, SBP, OB, SH, and RB are ubiquitous fibrous ingredients which had been extensively used in foods and feeds. Fermentation characteristics of DF for these fibrous ingredients in the hindgut are vital parts of the knowledge about the rational use of DF. Some studies tried to elucidate the fermentation characteristic of the DF contained in the fibrous feedstuffs and investigated the SCFA production in GIT by focusing on the fecal SCFA, even the ileal SCFA (23, 24). Nonetheless, short-chain fatty acids existing in the feces and ileal digesta could not represent the production of SCFA in GIT (25). The diet is digested and partially fermented by bacteria into SCFA in the upper tract after ingested by pigs. Part of the SCFA produced in the upper tract is absorbed by the host, and the others flowed into the hindgut accompanied by undigested nutrients. Short-chain fatty acids produced in the hindgut is derived from the ileal digesta fermentation by microbes in the hindgut. The SCFA in the hindgut was mainly absorbed by the host as the energy for the epithelium and enter the blood circulation, and just a small quantity of unabsorbed SCFA and unfermented nutrients were excreted to the outside of the body as feces. The amount of SCFA absorbed in the upper GIT was not accounted in this research (Figure 1).

In this research, an *in vivo–vitro* method was used to predict the SCFA production and absorption in the hindgut of growing pigs fed with diets containing WB, CB, SBP, OB, and SH, as the only fiber source, respectively. As the experimental setup, the upper tract digestion was conducted in the pig model, and *in vitro* fermentation trial was conducted to mimic the hindgut fermentation, where ileal digesta served as the substrate and incubated with pig fecal inoculum. By analyzing the combining result of *in vivo–vitro* trial, we could predict the SCFA production and absorption in the hindgut (26).

The source and solubility of DF are crucial determinants for the fermentation rate and the production of SCFA in the GIT. The SDF was easier to be utilized by microbiota due to its great hydrolysis capacity (27). The previous study researched the effects of SDF and IDF on predicted SCFA production and absorption in the hindgut. Flaxseed meal and oat hulls were used as the source of SDF and IDF, respectively, but this research did not confirm the uniqueness of fiber source in diet and neglected the interactions of different source fibers (15). The source of DF is not only affect the solubility of DF but also the composition of DF, which also affecting factors for SCFA production and absorption in the hindgut (28).

The high concentration of ileal acetate and propionate in SBP and SH groups might because of the high content of SDF in SBP and SH diets. SDF was fermented faster and more extensively by the microbiota compared with the IDF in the GIT (29). The low ileal SCFA but high content of SDF was also found in the OB group, which verified our hypothesis that solubility and source of DF were affecting the production of SCFA. The higher normalized concentration of fecal SCFA than ileal SCFA was also found in the research of Ndou et al. (15), but contrast to the research of Coles et al. (18). The reason could be the difference in the experimental model, as both Ndou's research and our research were conducted in a pig model, whereas the study of Coles et al. used human feces as the inoculum.

This research found that the predicted acetate and propionate production were the highest in SBP group but the lowest in OB group, since the ileal digesta of SBP and OB groups was fermented into the highest and lowest amount of SCFA by *in vitro* fermentation method, respectively. The great ability of acetate and propionate production of SBP fermentation has been mentioned in our past research, a significant positive correlation was found between cellulose and acetate (24), and the SBP group also had a high hindgut fermentability of cellulose in this research. Wang's research also found the inclusion of SBP caused high SCFA production (30). The low SCFA production capacity in the OB group was proved by the previous research where pigs fed with a 27% WB-based diet excreted more acetate, propionate, and total SCFA in feces compared with pigs fed with a diet containing 36% OB. Pigs fed OB diets inhibited the proliferation of *Succinivibrio* and *Prevotella*, which had been confirmed by a close relationship with fiber degradation and SCFA production (31). The lowest predicted apparent propionate and butyrate absorption in CB group were found in this research as well, and the low fermentability of CB fiber in the hindgut could be the main reason. Determination of fiber fermentability verified our inference that the CB group was found the lowest TDF, SDF, IDF, and DM fermentability in the hindgut. Meanwhile, propionate and butyrate production in the CB group were the lowest among all groups. The predicted total SCFA production in the hindgut in WB, CB, SBP, OB, SH, and RB group ranged 838–1,936 (mmol/kg DMI) in this study, whereas the hindgut SCFA production ability of pigs fed the diets containing wheat flour, WB and OB in Christensen et al. study ranged 369–850 (mmol/kg DMI) (26). Multiple fiber sources and high fiber content in diets could be the reason for the wide range of predicted SCFA production. Additionally, the higher body weight of pigs might also contribute to the variation of the range, as pigs with low body weight was found to have lower DF digestibility than pigs with high body weight, leading to high SCFA production in the hindgut (8).

Changes in predicted hindgut SCFA absorption and absorption were commonly consistent with each other. The high estimate of quantities of SCFA absorption in SBP group could be the result of the high quantity of SCFA production, since the SCFA in the large intestine was rapidly absorbed by the host through the passive diffusion way (32). The range of predicted apparent total SCFA absorption in this research was 89.64–96.86%, which was consistent with the previous study that colonocytes absorbed 90–95% of the SCFA produced in the humans' and pigs' hindgut (25).

The high hindgut DF fermentability and high level of SCFA production during fermentation (*in vitro*) were found in the SBP group. The high hindgut fermentability of SDF, NDF, ADF, and cellulose was found when growing pigs fed with diets containing CB, SH, and SBP (33). However, pigs in the OB group had the high hindgut DF fermentability as pigs fed with SBP, but the amount of SCFA production of pigs in OB group was less in this research. The reason might be the low IDF content and high SDF content in the OB diet. Microbes in the foregut had been proved the high DF degradation ability, and SDF could be fermented more rapidly by the gut microbes due to its high capacity for hydration compared to IDF (20). A large amount of SDF was degraded in the foregut, only a small amount of IDF was fermented into SCFA in the hindgut. Moreover, the predicted hindgut fermentability of DM (*in vitro*) was parallel to the determined hindgut DM fermentability (*in vivo*), which showed the credibility of *in vivo–vitro* method (22).

## Conclusion

Short-chain fatty acids in ileal digesta and feces cannot represent the ability of SCFA production. The DF sources had a great impact on SCFA production and absorption in the hindgut of growing pigs. The fiber of SBP showed the great ability of SCFA production and absorption with high SCFA concentration in ileal digesta and feces. Oat bran fiber showed a low potential for SCFA production and absorption even with high SCFA concentration and hindgut DF fermentability.

## Data Availability Statement

The original contributions presented in the study are included in the article/Supplementary Material, further inquiries can be directed to the corresponding author.

## Ethics Statement

The animal study was reviewed and approved by Laboratory Animal Welfare and Animal Experimental Ethical Inspection Committee in China Agricultural University.

## Author Contributions

YB and JW designed the study. YB, XZ, and JZ conducted the *vivo* and *vitro* experiments. YB analyzed data, performed the statistical analysis, and wrote the paper. ZW, HY, YP, DH, DC, SZ, and JW critically reviewed the manuscript. All authors have read and approved the final manuscript.

## Funding

This study was supported by the National Natural Science Foundation of China (31972596, 31630074), the Postdoctoral Innovative Talent Support Program (BX20200365), the Beijing Municipal Natural Science Foundation (S170001), and the China Scholarship Council (201913043).

## Conflict of Interest

YP was employed by company Boen Biotechnology Co. Ltd. The remaining authors declare that the research was conducted in the absence of any commercial or financial relationships that could be construed as a potential conflict of interest. The reviewer YZ declared a shared affiliation, with the authors to the handling editor at the time of the review.

## Publisher's Note

All claims expressed in this article are solely those of the authors and do not necessarily represent those of their affiliated organizations, or those of the publisher, the editors and the reviewers. Any product that may be evaluated in this article, or claim that may be made by its manufacturer, is not guaranteed or endorsed by the publisher.
