# Supplementary material for: Sources of Dietary Fiber Affect the SCFA Production and Absorption in the Hindgut of Growing Pigs
Source: Front Nutr. 2022 Jan 10;8:719935. doi: 10.3389/fnut.2021.719935 (PMC8784547; doi:10.3389/fnut.2021.719935)
Supplement: Supplementary file 1 [file Table_1.docx]

Supplementary Material

**Supplemental Table 1.** Ingredients and determined nutrient compositions of the growing pig diets

| Ingredients (%) |  |
| --- | --- |
| Corn | 81.82 |
| Soybean meal | 15.00 |
| Limestone | 0.75 |
| Dicalcium-phosohate | 0.65 |
| NaCl | 0.35 |
| *L*-lysine-HCl | 0.50 |
| DL-Methionine | 0.10 |
| L-Threonine | 0.15 |
| L-Tryptophan | 0.06 |
| L-Valine | 0.12 |
| Premix^1^ | 0.50 |
| Nutrient (% DM) |  |
| GE (MJ/kg DM) | 1.62 |
| CP | 17.86 |
| EE | 3.49 |
| NDF | 14.08 |
| ADF | 3.47 |

^1^ Premix: Vitamin A, 5512 IU/kg; vitamin D3, 2200 IU/kg; vitamin E, 64 IU/kg; vitamin K3, 2.2 mg/kg; vitamin B12/kg, 27.6 ug/kg; riboflavin, 5.5 mg/kg; pantothenic acid, 13.8 mg/kg; niacin, 30.3 mg/kg; choline chloride, 551 mg/kg; Mn (MnO), 40 mg/kg; Fe (FeSO_4_·H_2_O), 100 mg/kg; Zn (ZnO), 100 mg/kg; Cu (CuSO_4_·5H_2_O), 100 mg/kg; I (KI), 0.3 mg/kg; Se (Na_2_SeO_3_), 0.3 mg/kg. (The concentration of vitamins and trace minerals were showed in diet content style)

GE, gross energy; CP, crude protein; EE, ether extract; NDF, neutral detergent fiber; ADF, acid detergent fiber;

**Supplemental Table 2.** Ileal and fecal SCFA concentration for ileal cannulated pigs fed diets containing different sources of fiber (n = 6 per group)

|  | Diets^1^ | | | | | |  |  |
| --- | --- | --- | --- | --- | --- | --- | --- | --- |
| Items | WB | CB | SBP | OB | SH | RB | SEM^2^ | *P*-value |
| Determined ileal SCFA concentration (mmol/kg) | | | | | | | | |
| Acetate | 63.34 | 92.93 | 82.21 | 52.03 | 91.98 | 79.19 | 7.91 | > 0.05 |
| Propitiate | 18.15^b^ | 15.19^b^ | 5.01^b^ | 1.58^b^ | 43.30^a^ | 8.78^b^ | 2.87 | < 0.01 |
| Butyrate | 1.07^c^ | 2.79^abc^ | 2.29^bc^ | 6.34^a^ | 5.89^ab^ | 2.23^bc^ | 0.63 | < 0.01 |
| Total SCFA^2^ | 82.56^bc^ | 110.91^ab^ | 89.51^bc^ | 59.95^c^ | 141.16^a^ | 90.2^bc^ | 7.82 | < 0.01 |
| Determined Fecal SCFA concentration (mmol/kg) (*in vivo*) | | | | | | | | |
| Acetate | 113.53^cd^ | 124.56^bcd^ | 160.94^abc^ | 177.5^ab^ | 196.78^a^ | 98.82^d^ | 10.58 | < 0.01 |
| Propitiate | 45.60^b^ | 62.95^b^ | 58.34^b^ | 63.92^b^ | 137.65^a^ | 36.13^b^ | 9.73 | < 0.01 |
| Butyrate | 26.00^ab^ | 28.06^ab^ | 26.08^ab^ | 44.26^ab^ | 70.60^a^ | 11.62^b^ | 8.47 | < 0.05 |
| Total SCFA | 185.13^b^ | 215.57^b^ | 245.36^b^ | 285.68^ab^ | 405.03^a^ | 146.57^b^ | 26.77 | < 0.01 |

^1^ WB, wheat bran; CB, corn bran; SBP, sugar beet pulp; OB, oat bran; SH, soybean hulls; RB, rice bran.

^2^ Total SCFA = acetate + propionate + butyrate.

**Supplemental Table 3.** Nutrient compositions of fibrous ingredients

| Nutrient (%, DM) | WB | CB | SBP | OB | SH | RB |
| --- | --- | --- | --- | --- | --- | --- |
| TDF | 45.97 | 66.12 | 73.69 | 46.20 | 69.87 | 32.64 |
| SDF | 1.44 | 0.74 | 6.54 | 7.50 | 3.10 | 1.45 |
| IDF | 14.97 | 15.79 | 10.04 | 9.04 | 13.32 | 14.87 |
| NDF  ADF  Cellulose  Hemicellulose | 39.41  10.84  8.57  28.57 | 71.72  18.56  15.68  53.16 | 60.49  26.53  19.20  33.96 | 50.17  8.80  5.69  41.37 | 77.06  45.96  39.53  31.10 | 30.12  11.92  2.06  18.20 |

^1^ WB, wheat bran; CB, corn bran; SBP, sugar beet pulp; OB, oat bran; SH, soybean hulls; RB, rice bran.

TDF, total dietary fiber; SDF, soluble dietary fiber; IDF, insoluble dietary fiber; NDF, neutral detergent fiber; ADF, acid detergent fiber.
